# Inhibition of cAMP-Activated Intestinal Chloride Secretion by Diclofenac: Cellular Mechanism and Potential Application in Cholera

**DOI:** 10.1371/journal.pntd.0003119

**Published:** 2014-09-04

**Authors:** Pawin Pongkorpsakol, Nutthapoom Pathomthongtaweechai, Potjanee Srimanote, Sunhapas Soodvilai, Varanuj Chatsudthipong, Chatchai Muanprasat

**Affiliations:** 1 Department of Physiology, Faculty of Science, Mahidol University, Bangkok, Thailand; 2 Research Center of Transport Protein for Medical Innovation, Faculty of Science, Mahidol University, Bangkok, Thailand; 3 Graduate Study, Faculty of Allied Health Sciences, Thammasat University, Pathumtanee, Thailand; University at Buffalo, The State University of New York, United States of America

## Abstract

Cyclic AMP-activated intestinal Cl^−^ secretion plays an important role in pathogenesis of cholera. This study aimed to investigate the effect of diclofenac on cAMP-activated Cl^−^ secretion, its underlying mechanisms, and possible application in the treatment of cholera. Diclofenac inhibited cAMP-activated Cl^−^ secretion in human intestinal epithelial (T84) cells with IC_50_ of ∼20 µM. The effect required no cytochrome P450 enzyme-mediated metabolic activation. Interestingly, exposures of T84 cell monolayers to diclofenac, either in apical or basolateral solutions, produced similar degree of inhibitions. Analyses of the apical Cl^−^ current showed that diclofenac reversibly inhibited CFTR Cl^−^ channel activity (IC_50_∼10 µM) via mechanisms not involving either changes in intracellular cAMP levels or CFTR channel inactivation by AMP-activated protein kinase and protein phosphatase. Of interest, diclofenac had no effect on Na^+^-K^+^ ATPases and Na^+^-K^+^-Cl^−^ cotransporters, but inhibited cAMP-activated basolateral K^+^ channels with IC_50_ of ∼3 µM. In addition, diclofenac suppressed Ca^2+^-activated Cl^−^ channels, inwardly rectifying Cl^−^ channels, and Ca^2+^-activated basolateral K^+^ channels. Furthermore, diclofenac (up to 200 µM; 24 h of treatment) had no effect on cell viability and barrier function in T84 cells. Importantly, cholera toxin (CT)-induced Cl^−^ secretion across T84 cell monolayers was effectively suppressed by diclofenac. Intraperitoneal administration of diclofenac (30 mg/kg) reduced both CT and *Vibrio cholerae*-induced intestinal fluid secretion by ∼70% without affecting intestinal fluid absorption in mice. Collectively, our results indicate that diclofenac inhibits both cAMP-activated and Ca^2+^-activated Cl^−^ secretion by inhibiting both apical Cl^−^ channels and basolateral K^+^ channels in intestinal epithelial cells. Diclofenac may be useful in the treatment of cholera and other types of secretory diarrheas resulting from intestinal hypersecretion of Cl^−^.

## Author Summary

Diarrhea in cholera results from stimulation of cAMP-mediated intestinal Cl^−^ secretion by cholera toxin (CT). This study demonstrates that diclofenac, a widely used non-steroidal anti-inflammatory drug (NSAID), inhibited cAMP-activated Cl^−^ secretion in human intestinal epithelial (T84) cells by inhibiting both apical Cl^−^ channels (i.e. CFTR) and cAMP-activated basolateral K^+^ channels (i.e. KCNQ1/KCNE3). The mechanism by which CFTR was inhibited did not involve changes in intracellular cAMP levels and activation of negative regulators of CFTR activity including AMP-activated protein kinase (AMPK) and protein phosphatase. In addition, diclofenac suppressed two other types of apical Cl^−^ channels, namely, Ca^2+^-activated Cl^−^ channels and inwardly rectifying Cl^−^ channels, and Ca^2+^-activated basolateral K^+^ channels (i.e. K_Ca_3.1) without affecting Na^+^-K^+^ ATPase and Na^+^-K^+^-Cl^−^ cotransporter activities. Of particular importance, diclofenac at 30 mg/kg, which is the human equivalent dose for treatment of pain and inflammation (∼2 mg/kg in human), exhibited anti-secretory efficacy in mouse closed-loop models of cholera induced by either CT or *V. cholerae*. This study provides a rational basis for further development of diclofenac and related compounds as anti-diarrheal therapy for cholera and other types of diarrheas resulting from Cl^−^ transport-driven intestinal fluid secretion.

## Introduction

**Figure 1:**
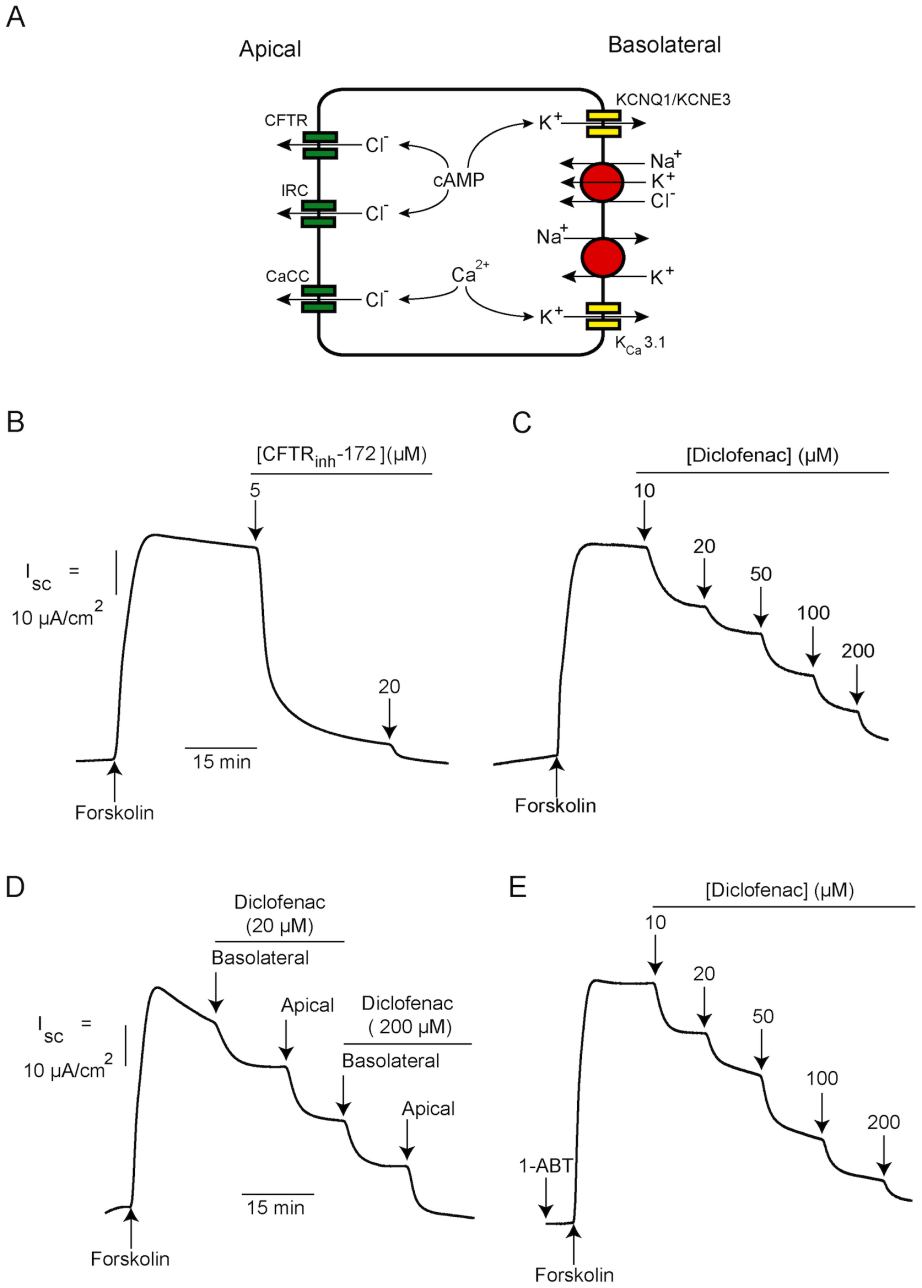
Effects of diclofenac on cAMP-activated Cl^−^ secretion in T84 cells. (A) A model of Cl^−^ secretion by an enterocyte showing transport proteins involved in transepithelial Cl^−^ secretion. CFTR, cystic fibrosis transmembrane conductance regulator; IRC, inwardly rectifying Cl^−^ channel; CaCC, Ca^2+^-activated Cl^−^ channel; KCNQ1/KCNE3, cAMP-activated K^+^ channel; K_Ca_3.1, Ca^2+^-activated K^+^ channel. (B) CFTR mediated cAMP-activated Cl^−^ secretion induced by forskolin. A representative short-circuit current tracing is shown (n = 5). (C) Effect of diclofenac on cAMP-activated Cl^−^ secretion. Diclofenac at the indicated concentrations was added into both apical and basolateral solutions (n = 4). (D) Polarity of inhibition by diclofenac on cAMP-activated Cl^−^ secretion. Diclofenac at the indicated concentrations was sequentially added into basolateral and apical solutions, respectively. A representative short-circuit current tracing is shown (n = 5). (E) The inhibition by diclofenac does not require metabolic activation. T84 cell monolayers were pretreated with 1-ABT, an inhibitor of CYP enzymes (1 mM). Diclofenac at the indicated concentrations was added into both apical and basolateral solutions. A representative short-circuit current tracing is shown (n = 5).

Transepithelial Cl^−^ secretion is an essential transport process in intestine and plays an important role in determining intestinal fluid secretion [1]. Chloride secretion creates a negative electrical potential, which in turn provides a driving force for transport of Na^+^ and water into intestinal lumen. Stimulation of Cl^−^ secretion by secretagogues (e.g. hormones, neurotransmitters, and enterotoxins) occurs mostly via cAMP or Ca^2+^-mediated pathways [2]. The Cl^−^ secretory process requires coordinated functions of several types of transport proteins located in both apical membrane (i.e. Cl^−^ channels) and basolateral membrane (i.e. Na^+^-K^+^-Cl^−^ cotransporters, Na^+^-K^+^ ATPases, and K^+^ channels) of enterocytes (Fig. 1A) [2], [3]. Both cAMP- and Ca^2+^-mediated Cl^−^ secretion require Na^+^-K^+^-Cl^−^ cotransporters (NKCC1) and Na^+^-K^+^ ATPases to take up Cl^−^ and maintain their driving force, respectively. In contrast, the apical chloride channels and basolateral K^+^ channels involved in the cAMP and Ca^2+^-mediated pathways are of distinct types. Cystic fibrosis transmembrane conductance regulator (CFTR) Cl^−^ channels and KCNQ1/KCNE3 K^+^ channels are involved in cAMP-activated Cl^−^ secretion, whereas Ca^2+^-activated Cl^−^ channels (CaCC) and K_Ca_3.1 K^+^ channels are involved in Ca^2+^-activated Cl^−^ secretion [3]–[6]. Interestingly, a recent study using human intestinal epithelial (T84) cells suggested that inwardly rectifying Cl^−^ channels (IRC) provided an alternative route for apical Cl^−^ exit during cAMP-activated Cl^−^ secretion [7]. Importantly, abnormal Cl^−^ secretion has been implicated in the pathogenesis of diseases. For example, decreased intestinal Cl^−^ secretion is associated with constipation in cystic fibrosis, while increased intestinal Cl^−^ secretion causes secretory diarrhea in cholera and Traveler's diarrhea (caused by enterotoxigenic *Escherichia coli*) [8].

Cholera is a severe type of secretory diarrhea resulted from intestinal infection with *Vibrio cholera* and kills hundreds of thousand people per year [9]–[11]. At present, the mainstay therapy of cholera is the use of oral rehydration solution (ORS), which is effective only in 80% of cholera cases [9]. However, ∼20% of cholera patients require intravenous fluid replacement because their intestinal fluid loss is too severe to be replenished by ORS [9], [12]. Diarrhea in cholera is known to result mainly from the pro-secretory effect of cholera toxin (CT) produced by *V. cholera* on enterocytes [12]. After internalization into enterocytes, cholera toxins induce an elevation of intracellular cAMP and subsequent CFTR-dependent Cl^−^ secretion, resulting in intestinal fluid secretion and fluid loss [12]. With an attempt to develop anti-secretory therapy of cholera, several classes of CFTR inhibitors have been identified and demonstrated to effectively reduce CT-induced intestinal fluid secretion in both rats and mice [13]–[16]. Interestingly, a recent study using a *V. cholerae* infection model in adult mice confirmed CFTR as a major host factor determining intestinal fluid secretion in cholera [17]. Accordingly, CFTR is regarded as a promising drug target for cholera.

Non-steroidal anti-inflammatory drugs (NSAIDs), a group of commonly used drugs exerting their anti-inflammatory action via inhibition of cyclooxygenases, have been shown to be functional modulators of both cation and anion channels in various types of tissues [18]. Interestingly, ibuprofen and fenamates such as flufenamic acid have been shown to inhibit CFTR in respiratory epithelial cells and in *Xenopus* oocytes, respectively [19], [20]. However, the effects of another widely used and better-tolerated cyclooxygenase 2 (COX-2)-selective NSAID, diclofenac, on epithelial Cl^−^ channels including CFTR remain unexplored. Indeed, this drug has been shown to directly inhibit several types of cation channels including acid sensing ion channels (ASIC), voltage-sensitive sodium channels, and transient receptor potential (TRP) channels [18], [21]. Since diclofenac shares similarity in chemical structure and spectrum of activity against some ion channels (especially ASIC and TRP channels) with flufenamic acid and ibuprofen, we hypothesized that diclofenac may inhibit CFTR and reduce cAMP-activated Cl^−^ secretion in intestinal epithelia. Therefore, this study was performed to investigate the effect of diclofenac on cAMP-activated intestinal Cl^−^ secretion and its underlying mechanisms using T84 cell monolayers as a model of intestinal epithelia. In addition, potential utility of diclofenac in the treatment of cholera was investigated *in vivo* using the two mouse closed-loop models of cholera induced by CT and by *V. cholerae*.

## Methods

### Ethics statement

This study has been approved by the Institutional Animal Care and Use Committee of the Faculty of Science, Mahidol University (permit number MUSC56-022-284). This study was performed in accordance with the recommendations in the Guide for the Care and Use of Laboratory Animals of the US National Institutes of Health.

### Chemicals

CFTR_inh_-172 was obtained from Calbiochem (San Diego, California, USA), cholera toxin was from List Biological Laboratories, Inc. (Campbell, California, USA) whereas trypsin-EDTA, fetal bovine serum, penicillin and streptomycin were from HyClone (Logan, Utah, USA). Other chemicals were obtained from Sigma-Aldrich (St. Louis, Missouri, USA).

### Cell culture

T84 cells and Calu-3 cells were obtained from American Type Culture Collection (Manassas, Virginia, USA). T84 cells were cultured in 50% Dulbecco's Modified Eagle's medium and 50% Ham's F-12 medium supplemented with 10% fetal bovine serum, in the presence of 100 U/ml penicillin and 100 µg/ml streptomycin. Calu-3 cells were cultured in Eagle's Minimum Essential Medium supplemented with 10% fetal bovine serum, 100 U/ml penicillin, and 100 µg/ml streptomycin. Both types of cells were maintained at 37°C in a humidified incubator under an atmosphere of 95% O_2_/5% CO_2_. For electrophysiological analysis, T84 cells and Calu-3 cells were plated onto Snapwell inserts at a density of 5×10^5^ cells/well, and grown in a humidified incubator with daily replacement of culture media for 14 days.

### Electrophysiological analysis

In these experiments, inserts (with transepithelial electrical resistance >1,000 Ω.cm^2^ as measured by EVOM2 volt-ohm meter (World Precision Instruments, Sarasota, Florida, USA)) were mounted in Ussing chambers. For short-circuit current (I_sc_) measurements, both apical and basolateral hemichambers were filled with Kreb's solution containing (pH 7.3) 120 mM NaCl, 25 mM NaHCO_3_, 3.3 mM KH_2_PO_4_, 0.8 mM K_2_HPO_4_, 1.2 mM MgCl_2_, 1.2 mM CaCl_2_ and 10 mM glucose. For I_sc_ analysis of mouse intestine, a sheet of mouse ileum was prepared without muscle stripping and mounted in Ussing chambers filled with Kreb's solutions containing indomethacin (10 µM in both apical and basolateral solutions; to prevent prostaglandin-induced Cl^−^ secretion) and amiloride (10 µM in apical solution; to prevent current contributed by Na^+^ absorption). For apical Cl^−^ current measurements, apical and basolateral hemichambers were filled with low Cl^−^ and high Cl^−^ solutions, respectively, to create a basolateral-to-apical Cl^−^ gradient. High Cl^−^ basolateral solution contained (pH 7.3) 130 mM NaCl, 2.7 mM KCl, 1.5 mM KH_2_PO_4_, 1 mM CaCl_2_, 0.5 mM MgCl_2_, 10 mM Na-HEPES and 10 mM glucose. In low Cl^−^ apical solution, 65 mM NaCl was replaced with 65 mM sodium gluconate, and the concentration of CaCl_2_ was increased to 2 mM. To induce basolateral membrane permeabilization, amphotericin B (250 µg/ml) was added into basolateral solutions and incubated for 30 min prior to apical Cl^−^ current measurements.

For basolateral K^+^ current measurements, apical and basolateral hemichambers were filled with high K^+^ and low K^+^ solutions, respectively, to establish an apical-to-basolateral K^+^ gradient. High K^+^ apical solution contained (pH 7.3) 142.5 mM K-gluconate, 1.25 mM CaCl_2_, 0.40 mM MgSO_4_, 0.43 mM KH_2_HPO_4_, 0.35 mM Na_2_HPO_4_, 10 mM Na-HEPES and 5.6 mM glucose. In low K^+^ basolateral solution, concentration of K-gluconate was reduced to 5.4 mM and 136.9 mM *N*-methyl-glucamine was added. Before basolateral K^+^ current measurements, apical membrane of T84 cells was permeabilized by amphotericin B (250 µg/ml) and ouabain (1 mM) was added into basolateral solution to prevent current contributed by Na^+^-K^+^ ATPase. In the measurement of Ca^2+^-activated basolateral K^+^ channel activity, BaCl_2_ (5 mM) was added into the basolateral solution to prevent current contributed by cAMP-activated basolateral K^+^ channels.

For determination of Na^+^-K^+^ ATPase activity, both apical and basolateral hemichambers were filled with Kreb's solutions. After initiating I_sc_ measurements, cells were treated with DMSO (control) or diclofenac (added into basolateral solution), followed by apical membrane permealization by amphotericin B (250 µg/ml). After stabilization of the amphotericin B-induced rise in I_sc_, ouabain (1 mM) was added into the basolateral solution and the values of ouabain-sensitive current were used to represent the activity of Na^+^-K^+^ ATPases.

Kreb's solutions were bubbled continuously with 95% O_2_/5% CO_2_ whereas Cl^−^ and K^+^ solutions were bubbled with 100% O_2_. All solutions were maintained at 37°C. Short-circuit current/apical Cl^−^ current/basolateral K^+^ current/Na^+^-K^+^-mediated current was recorded using a DVC-1000 voltage-clamp (World Precision Instruments, Sarasota, Florida, USA) with Ag/AgCl electrode and 3 M KCl agar bridge.

### Intracellular cAMP measurement

Intracellular cAMP levels in T84 cells were measured using cAMP immunoassays (R&D Systems, Minneapolis, Minnesota, USA). T84 cells were seeded on 24-well plates at a density of 10^6^ cells/well and grown for 24 h in a humidified 5% CO_2_/95% O_2_ incubator at 37°C. Then, cells were washed three times with PBS before 1-h treatments with DMSO (vehicle), diclofenac (200 µM), forskolin (20 µM), or forskolin (20 µM) plus diclofenac (200 µM). Thereafter, cells were lysed with cell lysis buffer and level of intracellular cAMP was measured according to the manufacturer's instructions.

### Assay of Na^+^-K^+^-Cl^−^ cotransporter activity

N^+^-K^+^-C1^−^ cotransporter (NKCCl) activity in T84 cells was measured using Thallium (Tl^+^) influx-based fluorescent assays (Invitrogen, Carlsbad, California, USA) with some modifications [22]. Briefly, T84 cells were seeded on 96-well plates at a density of 10^5^ cells/well and grown for 48 h in a humidified 5% CO_2_/95% O_2_ incubator at 37°C. Forty-eight hours later, culture media were removed and cells were incubated for 2 h with Cl^−^-free buffer containing fluorogenic Tl^+^-sensitive dye and probenecid (an organic anion inhibitor used to inhibit transporter-mediated dye efflux). Cells were then washed twice with Cl^−^-free buffer and incubated for 15 min with Cl^−^-free buffer containing Thallium sulfate (Tl_2_SO_4_, 5 mM) and clotrimazole (an inhibitor of basolateral K^+^ channels; 30 µM) with or without diclofenac (200 µM). Bumetanide (100 µM) was used as a positive control. For measurements of NKCC1 activity, fluorescent intensity (excitation wavelength = 490 nm; emission wavelength = 520 nm) was recorded 15 s before automated addition of NaCl solution (final [NaCl] = 135 mM) and thereafter for 30 s using a Wallac Victor^2^ microplate reader (Perkin Elmer, Waltham, Massachusetts, USA). NKCC1 activity was analyzed from the slope of linear increase in fluorescent intensity within 15 s following NaCl addition.

### Western blot analysis

T84 cells plated on 6 well-plates were incubated for 20 min with vehicle (DMSO), ATP (100 µM) or ATP (100 µM) plus diclofenac (20 µM). Proteins were extracted using lysis buffers containing (pH 7.4) 1% Triton X-100, 50 mM Tris-HCl, 150 mM NaCl, 1 mM EDTA, 1 mM NaF, 1 mM Na_3_VO_4_, 1 mM PMSF and protease inhibitor (PI) cocktail. Protein concentration was determined using a Lowry method. Equal amounts of proteins were loaded on sodium dodecyl sulfate polyacrylamide gel electrophoresis and transferred to a nitrocellulose membrane. The membrane was blocked with 0.5% milk for 90 min at room temperature before incubation overnight at 4°C with antibodies against phosphorylated Ca^2+^/calmodulin-dependent protein kinase II (CaMKII) or β-actin (Cell signaling technology, Denver, Colorado, USA). The secondary antibodies were horseradish peroxidase-conjugated anti-rabbit IgG antibodies. The immunoblot was visualized using a chemiluminescence detection method.

### Assay of cell viability

T84 cell viability was measured using 3-(4,5-dimethyl-2-thiazolyl)-2,5-diphenyl-2H-tetrazolium bromide (MTT) assays. In brief, T84 cells were plated on 96-well plates at a density of 1×10^5^ cells/well and grown overnight, followed by 24-h incubation with culture media containing DMSO (control) or diclofenac at various concentrations. After removal of culture media, cells were treated with MTT reagent (5 mg/ml) for 4 h at 37°C and the reaction was stopped by addition of an aliquot of DMSO (100 µl) into each well. Thirty minutes later, an absorbance at 540 nm was determined using a spectrophotometer.

### Barrier function assay

Barrier function of T84 cell monolayers was measured using fluorescein isothiocyanate (FITC)-labeled dextran (molecular weight ∼4.4 kDa) flux assays. Briefly, T84 cells were plated on Transwell membrane support (Costar, Cambridge, Massachusetts, USA) at a density of 5×10^5^ cells/well and grown for 14 days, when transepithelial electrical resistance was >1,000 Ω.cm^2^. Cells were then treated for 24 h with DMSO (control), diclofenac (20 µM and 200 µM), and EGTA (3 mM) (added to both apical and basolateral sides). To measure FITC-dextran flux, FITC-dextran (1 mg/ml) was added to apical side, and an hour later, basolateral media were sampled for measuring concentrations of FITC-dextran using Wallac Victor^2^ microplate reader (Perkin Elmer, Waltham, Massachusetts, USA).

### Mouse models of cholera and intestinal fluid absorption assays

To investigate the *in vivo* effect of diclofenac on CT- and *V. cholerae*-induced intestinal fluid secretion, mice (30–35 g, ICR strain; The National Laboratory Animal Center, Salaya, Nakornpathom, Thailand) were fasted for 24 h before experiments. After anesthesia by an intraperitoneal injection of thiopenthal sodium (50 mg/kg), an abdominal incision was made and 3 closed ileal loops (2–3 cm in length) were made by ligations and then instilled with 100 µl of phosphate-buffered saline (PBS) or PBS containing CT (1 µg) or *V. cholerae* (classical O1 569B strain of *V. cholerae* at 10^7^ CFU/loop). This strain of *V. cholerae* was used since it has been known to produce large amounts of CT and cause consistent intestinal fluid secretion in adult mouse closed-loop models [17]. Body temperature of mice was maintained at 36–37°C for the entire period of operation using heating pads. After abdominal closure by sutures, mice were intraperitoneally administered with DMSO (control) or diclofenac (30 mg/kg), and allowed to recover from anesthesia. Four hours (for experiments using CT) or 12 hours (for experiments using *V. cholerae*) later, mice were anesthetized again, abdomen was opened and ileal loops were removed for measurements of intestinal fluid secretion from loop weight/length ratios. Then, mice were euthanized with an injection of thiopenthal sodium (150 mg/kg). To determine the effect of diclofenac on intestinal fluid absorption, mouse ileal loops were instilled with PBS, with or without intraperitoneal administration of diclofenac (30 mg/kg). Ileal loops were removed for measuring loop weight/length ratios at 20 min and 40 min after PBS instillation.

### Statistical analysis

Results are presented as means ± S.E.M. Statistical differences between control and treatment groups were evaluated using Student's t test or one-way ANOVA followed by Bonferroni's post hoc test, where appropriate, with p value<0.05 being considered statistically significant.

## Results

### Effect of diclofenac on cAMP-activated Cl^−^ secretion

Cyclic AMP-activated Cl^−^ secretion across human intestinal epithelial (T84) cell monolayers was investigated using I_sc_ measurements. First, we investigated the relative contribution of CFTR to cAMP-activated Cl^−^ secretion in T84 cells. As shown in Fig. 1B, CFTR_inh_-172, a CFTR-specific Cl^−^ channel blocker, completely abolished the cAMP-activated Cl^−^ secretion elicited by forskolin (an adenylate cyclase activator), indicating that CFTR provided a primary route for apical Cl^−^ exit upon cAMP stimulation in these cells. Next, the effect of diclofenac on cAMP-activated Cl^−^ secretion was investigated. As depicted in Fig. 1C, diclofenac, concomitantly added into both apical and basolateral solutions, inhibited cAMP-activated Cl^−^ secretion in a concentration-dependent fashion, with an IC_50_ of ∼20 µM and almost complete inhibition at 200 µM. In addition, the polarity of inhibition by diclofenac was determined using a protocol of sequential additions of the drug. In this experiment, diclofenac was sequentially added into basolateral and apical solutions, respectively. As demonstrated in Fig. 1D, basolateral or apical additions of diclofenac (at final concentrations of 20 µM and 200 µM) produced a similar degree of inhibitions, suggesting that diclofenac equally affected both apical and basolateral transport processes. Since diclofenac is metabolized by intestinal cytochrome P450 (CYP) enzymes to hydroxylated diclofenac metabolites and reactive intermediates [23], we then investigated whether the inhibitory effect of diclofenac required metabolic activation by CYP enzymes. Figure 1E shows that pretreatment with 1-aminobenzotriazole (1-ABT; 1 mM) [24], an inhibitor of CYP enzymes, had virtually no effect on the inhibition of cAMP-activated Cl^−^ secretion in T84 cells, indicating that the effect of diclofenac did not require metabolic activation.

### Effect of diclofenac on CFTR-mediated apical Cl^−^ transport

**Figure 2:**
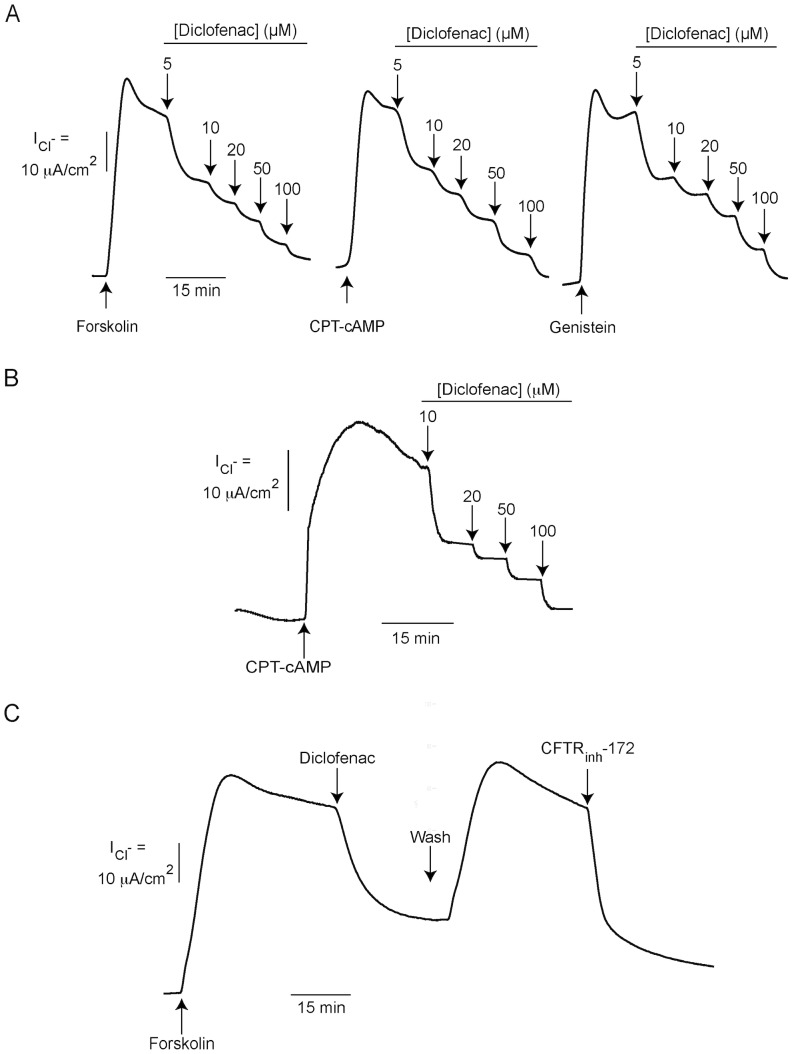
Effect of diclofenac on CFTR Cl^−^ channel activity. (A) Effect of diclofenac on CFTR Cl^−^ channel activity stimulated by forskolin (20 µM), CPT-cAMP (100 µM), or genistein (20 µM) in T84 cells. In this experiment, CFTR Cl^−^ channel activity was determined using apical Cl^−^ current analysis of T84 cell monolayers. Amphotericin B (250 µg/ml) was added into basolateral solutions to permeabilize basolateral membrane of T84 cells. Diclofenac at the indicated concentrations was added to both apical and basolateral sides. Representative current tracings of 4–7 experiments were shown. (B) Effect of diclofenac on CFTR Cl^−^ channel activity in human airway epithelial (Calu-3) cells. Diclofenac at the indicated concentrations was added into both apical and basolateral solutions after stimulation of CFTR-mediated apical Cl^−^ current by CPT-cAMP (100 µM). Apical Cl^−^ current analysis was performed with basolateral membrane of Calu-3 cells being permeabilized with amphotericin B (250 µg/ml). A representative current tracing of 5 separate experiments is shown. (C) Reversibility of inhibition of CFTR Cl^−^ channel activity by diclofenac (20 µM) in T84 cells. Apical Cl^−^ current analysis was performed with basolateral membrane permeabilization by amphotericin B. After stabilization of diclofenac -inhibited apical Cl^−^ current, solutions containing forskolin and diclofenac were removed, and hemichambers were gently washed three times and filled with fresh solutions containing only forskolin. At the end of experiment, CFTR_inh_-172 (5 µM) was added into apical solutions. A representative current tracing of 4 separate experiments is shown.

To investigate the effect of diclofenac on CFTR Cl^−^ channel activity, apical Cl^−^ current measurements were performed in T84 cells. In this experiment, basolateral membrane was permeabilized by amphotericin B and a Cl^−^ gradient was established using asymmetrical Cl^−^ buffers ([Cl^−^] in basolateral solution >[Cl^−^] in apical solution). Apical Cl^−^ current induced by CFTR agonists under this experimental condition, therefore, indicates CFTR Cl^−^ channel activity. As shown in Fig. 2A, apical Cl^−^ current induced by forskolin (20 µM), CPT-cAMP (cell-permeable cAMP; 100 µM), and genistein (direct CFTR activator; 20 µM) were inhibited by diclofenac in a dose-dependent manner, with IC_50_ of 8 µM, 8.5 µM and 10 µM, respectively, and with almost complete inhibition at 100 µM. These results suggest that diclofenac inhibited cAMP-activated Cl^−^ secretion in T84 cells, at least in part, by inhibiting CFTR Cl^−^ channel activity. To examine the effect of diclofenac on another cell type expressing human CFTR, apical Cl^−^ current analysis was performed using monolayers of Calu-3 cells, a human airway epithelial cell line. It was found that diclofenac also inhibited CFTR-mediated apical Cl^−^ current induced by CPT-cAMP in this cell line with IC_50_ of ∼10 µM and with near complete inhibition at 100 µM (Fig. 2B). In addition, the reversibility of diclofenac inhibition of CFTR Cl^−^ channel activity was investigated using apical Cl^−^ current measurements in T84 cells. The inhibitory effect of diclofenac on CFTR-mediated apical Cl^−^ current disappeared after removing diclofenac (20 µM) from bathing solutions (Fig. 2C), suggesting that the effect is reversible. Of note, the recovery of apical Cl^−^ current was inhibited by CFTR_inh_-172, confirming that the current was indeed CFTR-mediated Cl^−^ current.

### Mechanism of inhibition of CFTR-mediated Cl^−^ transport by diclofenac in T84 cells

**Figure 3:**
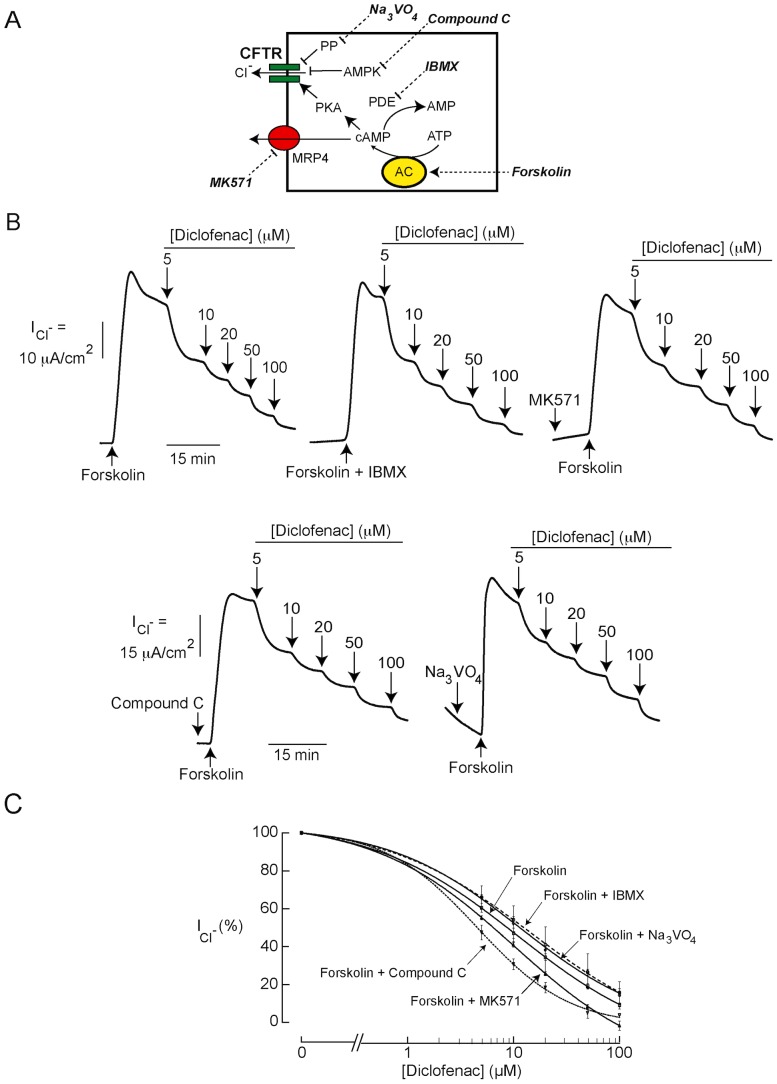
Mechanism of CFTR inhibition by diclofenac in T84 cells. (A) Schematic diagram illustrating the regulatory mechanisms of CFTR Cl^−^ channel activity in T84 cells. Inhibitors/activators used in this study are shown. PP, protein phosphatase; AMPK, AMP-activated protein kinase; PDE, phosphodiesterase; PKA, protein kinase A; AC, adenylate cyclase; MRP4, multidrug resistance-associated protein 4 (B) Involvements of PDE, MRP4, AMPK and protein phosphatase in the inhibition of CFTR-mediated Cl^−^ transport. Dose-inhibition studies of diclofenac were performed after the indicated treatments. Representative current tracings are shown. (C) Summary of dose-inhibition studies. Data are fitted to Hill equation and expressed as means of % agonist-stimulated apical Cl^−^ current ± S.E.M. (n = 6–8).

**Figure 4:**
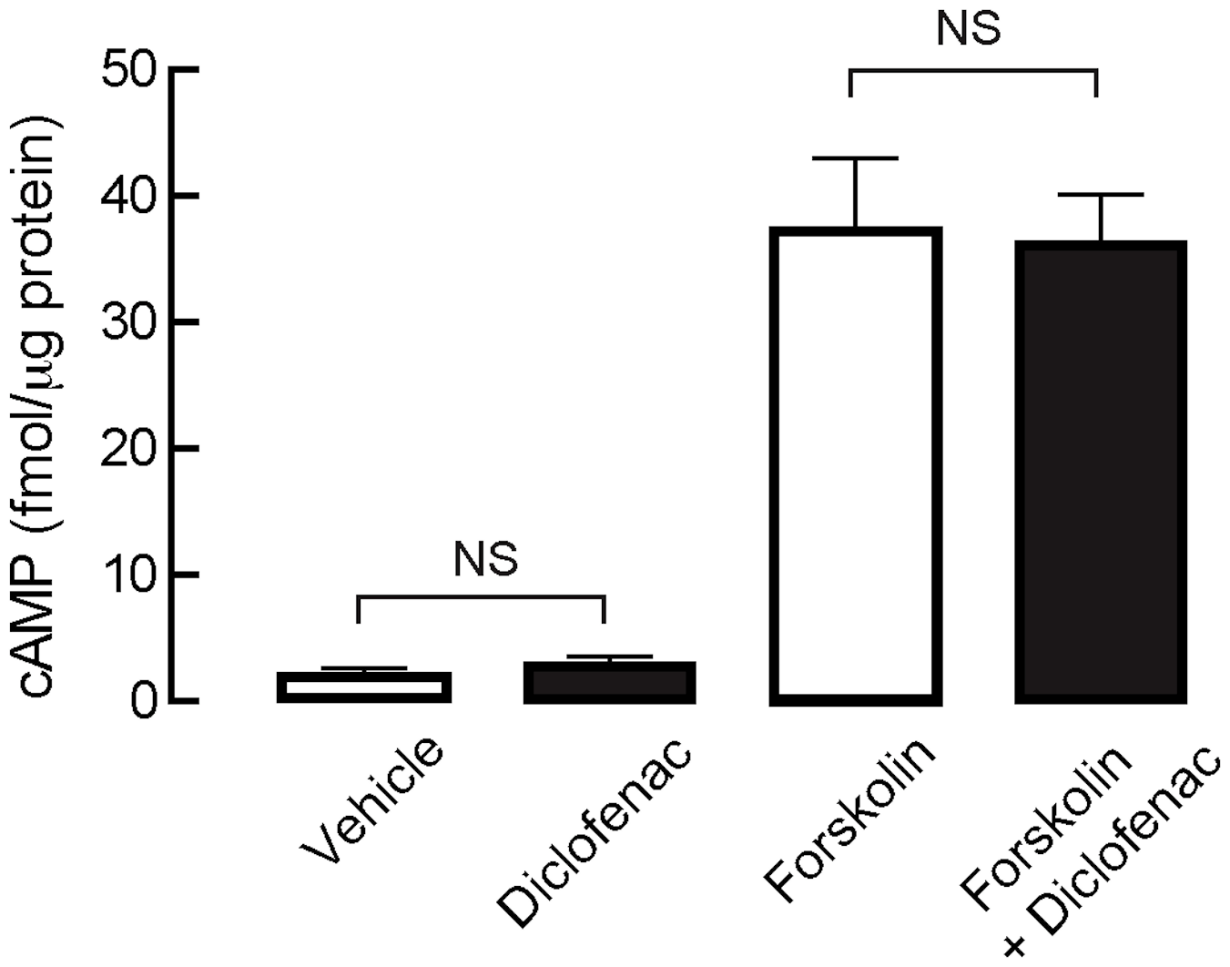
Effect of diclofenac on intracellular cAMP content in T84 cells. T84 cells were incubated for an hour with DMSO (vehicle), diclofenac (200 µM), forskolin (20 µM) or forskolin (20 µM) plus diclofenac (200 µM), followed by cell lysis and cAMP measurement using cAMP immunoassay kit. Data are expressed as means ± S.E.M. NS, non-statistical difference (n = 3).

Based on the finding that, in T84 cells, CFTR-mediated apical Cl^−^ current was effectively inhibited by diclofenac, subsequent experiments were performed to investigate the mechanisms by which CFTR Cl^−^ channel activity was suppressed in these cells using apical Cl^−^ current analysis. Figure 3A demonstrates the mechanisms of CFTR regulation in T84 cells. In general, CFTR Cl^−^ channel activity is regulated by cAMP-dependent protein kinase A (PKA) and AMP-activated protein kinase (AMPK) [25]. Phosphorylation at CFTR's R-domain by PKA and AMPK causes activation and inhibition of CFTR Cl^−^ channel activity, respectively [26], [27]. Levels of PKA phosphorylation at CFTR's R domain depends on activities of protein phosphatases [28], which dephosphorylate CFTR, and intracellular cAMP level, which stimulates PKA activities. On the other hand, intracellular cAMP level in T84 cells depends on the activities of adenylate cyclase (which generates cAMP), phosphodiesterase (PDE; which degrades cAMP) and multidrug resistance-associated protein 4 (MRP4; which mediates cAMP efflux) [12], [29]. Because the potencies of diclofenac inhibition of forskolin- and CPT-cAMP-stimulated apical Cl^−^ current in T84 cells were comparable (Fig. 2A), we hypothesized that the targets of diclofenac might be downstream to cAMP generation or involve AMPK activation. To investigate whether diclofenac indirectly inhibited CFTR by decreasing cAMP levels via activation of PDE or MRP4, activating AMPK, or stimulating protein phosphatases, dose-inhibition studies were performed in the presence or absence of inhibitors of these regulatory proteins. As demonstrated in Fig. 3B, the potency of inhibition of forskolin-induced Cl^−^ current in the presence of IBMX (PDE inhibitor; top-middle current tracing) was not different from that of control (CFTR Cl^−^ channel activity stimulated by forskolin; top-left current tracing), indicating that the inhibitory effect was not due to stimulation of PDE. Likewise, pretreatment with MK571 (MRP4 inhibitor; Fig. 3B, top-right current tracing), compound C (AMPK inhibitor; Fig. 3B, bottom-left current tracing) or Na_2_VO_3_ (protein phosphatase inhibitor; Fig. 3B, bottom-right current tracing) did not alter the potency of diclofenac. Range of the agonist-induced apical Cl^−^ current in these experiments was ∼40–60 µA/cm^2^. The summary of dose-inhibition studies is shown in Fig. 3C. Furthermore, the effect of diclofenac on intracellular cAMP contents was investigated in T84 cells using cAMP immunoassay kits. As depicted in Fig. 4, diclofenac at 200 µM, a concentration found to fully inhibit cAMP-activated Cl^−^ secretion in T84 cells, had virtually no effect on intracellular cAMP levels under both basal and forskolin-stimulated conditions. All together, the results suggest that the inhibition of CFTR Cl^−^ channel activity by diclofenac in T84 cells was not due to indirect mechanisms including an alternation of intracellular cAMP levels, AMPK activation, and CFTR dephosphorylation.

### Effect of diclofenac on cAMP-activated basolateral K^+^ channels, Na^+^-K^+^ ATPases and Na^+^-K^+^-Cl^−^ cotransporters in T84 cells

**Figure 5:**
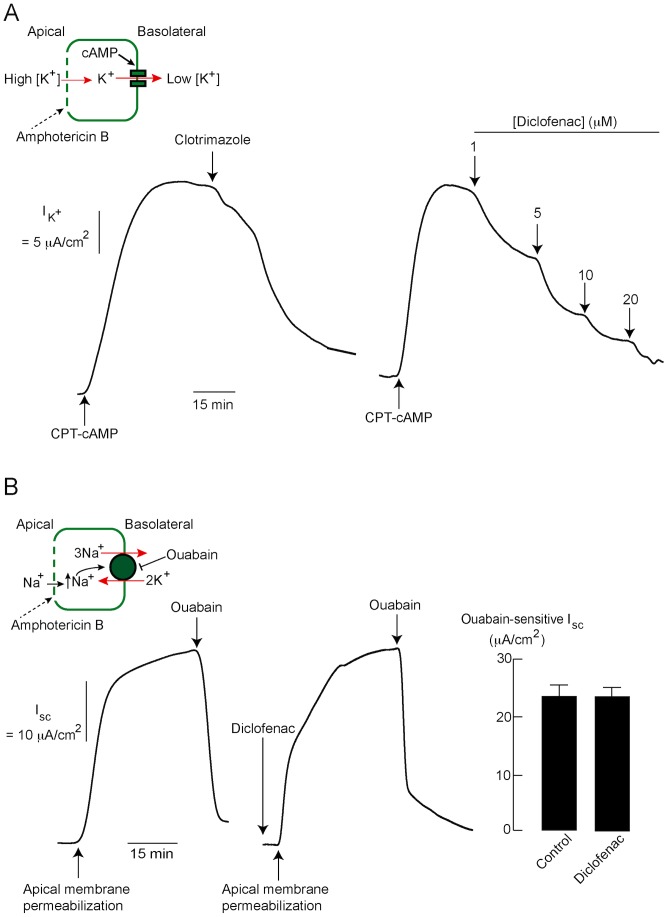
Effects of diclofenac on cAMP-activated basolateral K^+^ channels and Na^+^-K^+^ ATPases in T84 cells. (A) Inhibition of cAMP-activated basolateral K^+^ channels by diclofenac. Activity of cAMP-activated basolateral K^+^ channels was measured using basolateral K^+^ current analysis in T84 cells. Inset shows the condition for basolateral K^+^ current analysis. Representative basolateral K^+^ current tracings are shown; (left) validation of the method by clotrimazole, an inhibitor of cAMP-activated K^+^ channels, (right) dose-inhibition studies of the effect of diclofenac on cAMP-activated basolateral K^+^ current (n = 5). (B) No effect of diclofenac on Na^+^-K^+^ ATPase activity. Na^+^-K^+^ ATPase activity was estimated from values of ouabain-sensitive short-circuit current, which was induced by intracellular Na^+^ loading following amphotericin B (250 µg/ml)-induced apical membrane permeabilization. Inset shows the condition for measuring Na^+^-K^+^ ATPase activity. Representative short-circuit current tracings and summary of data are shown. Summary of data is expressed as means of ouabain-sensitive short-circuit current ± S.E.M. (n = 6–8).

Since additions of diclofenac into apical and basolateral solutions produced similar degree of inhibitory effects on cAMP-induced Cl^−^ secretion in T84 cells, we hypothesized that this drug may affect basolateral transport proteins, namely cAMP-activated K^+^ channels, Na^+^-K^+^ ATPases and Na^+^-K^+^-Cl^−^ cotransporters (NKCC1). The effect of diclofenac on cAMP-activated basolateral K^+^ channels was, therefore, investigated using basolateral K^+^ current analysis. In this method, apical membrane was permeabilized by amphotericin B (250 µg/ml) and a K^+^ gradient ([K^+^]_apical_>[K^+^]_basolateral_) was established using asymmetrical K^+^ buffers in the presence of ouabain in basolateral solutions (to prevent current generated by Na^+^-K^+^ ATPases) (Fig. 5A, inset). The basolateral K^+^ current elicited by an addition of cell-permeable cAMP (e.g. CPT-cAMP) would, therefore, reflect the activity of cAMP-activated K^+^ channels located in the basolateral membrane of T84 cells (i.e. KCNQ1/KCNE3 K^+^ channels). As shown in Fig. 5A (left), clotrimazole (30 µM), a known inhibitor of basolateral K^+^ channels, markedly inhibited the CPT-cAMP-induced current, validating this method for assessing the activity of cAMP-activated basolateral K^+^ channels. Interestingly, the cAMP-activated basolateral K^+^ current was dose-dependently inhibited by diclofenac with an IC_50_ of ∼3 µM and almost complete inhibition at 20 µM (Fig. 5A (right)). In addition, the effect of diclofenac on Na^+^-K^+^ ATPase was investigated using a protocol designed to specifically measure Na^+^-K^+^ ATPase activity. In this protocol, intracellular Na^+^ loading by amphotericin B-induced permeabilization of apical membrane stimulated Na^+^-K^+^ ATPase activity, resulting in an increase in I_sc_ (Fig. 5B, inset). Values of I_sc_ sensitive to ouabain (1 mM; added into basolateral solution), an inhibitor of Na^+^-K^+^ ATPases, were used as indicators of Na^+^-K^+^ ATPase activity. As depicted in Fig. 5B, pretreatment with diclofenac (200 µM) had no effect on ouabain-sensitive I_sc_, indicating that diclofenac had no effects on Na^+^-K^+^ ATPase activity.

**Figure 6:**
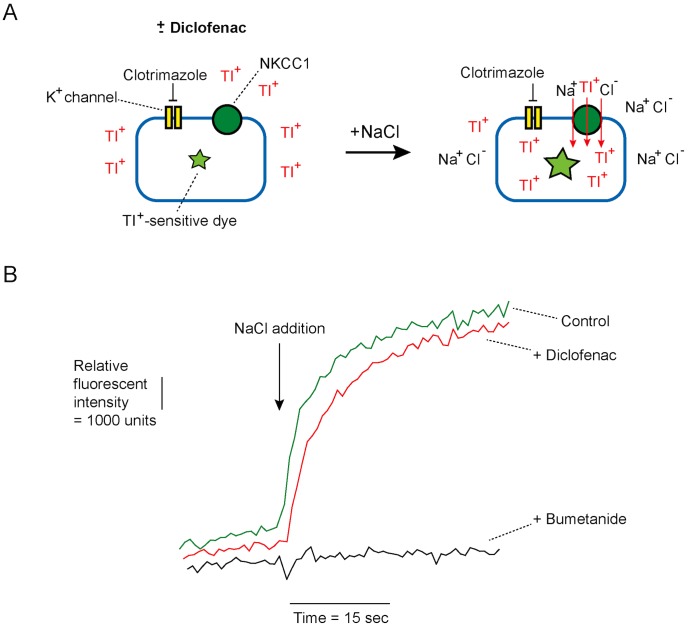
Effect of diclofenac on NKCC1 activity in T84 cells. (A) Diagrammatic protocol for Tl^+^ influx-based assay of NKCC1 activity. After loading with Tl^+^-sensitive dye, T84 cells were incubated for 15 min in the Cl^−^-free buffer containing Tl_2_SO_4_ and clotrimazole (to block K^+^ channels) with or without diclofenac (200 µM). Then, NaCl solution (final concentration of NaCl = 135 mM) was added to stimulate NKCC1-mediated Tl^+^ influx causing an increase in fluorescence from Tl^+^-sensitive dye. NKCC1 activity was analyzed from the slope of linear increases in fluorescent intensity within 15 s after NaCl addition. (B) Representatives of relative fluorescent signals from 5 separate experiments, without (control) and with diclofenac (200 µM). Bumetanide (100 µM), a known inhibitor of NKCC1, was used as a positive control.

Subsequently, the effect of diclofenac on NKCC1 activity was investigated in T84 cells using Thallium (Tl^+^) influx-based fluorescent assays [22]. In this experiment, cells were loaded with Tl^+^-sensitive dye and bathed in Cl^−^ -free bathing solution containing Tl^+^ and clotrimazole (30 µM; to prevent influx of Tl^+^ through K^+^ channels) (Fig. 6A). To measure NKCC1 activity, NaCl solution, which triggers NKCC1-mediated Tl^+^ uptake, was added into each well resulting in an increase in fluorescent intensity. NKCC1 activity was deduced from the slope of increased fluorescent signal within 15 s after NaCl addition. As depicted in Fig. 6B, pretreatment with diclofenac (200 µM) had no effect on Tl^+^ influx into T84 cells compared to control, whereas bumetanide (a known inhibitor of NKCC1; 100 µM) completely prevented Tl^+^ influx into these cells. These results indicate that the inhibitory effect of diclofenac on cAMP-activated Cl^−^ secretion was not through the inhibition of NKCC1.

### Effect of diclofenac on CaCC, IRC and Ca^2+^-activated basolateral K^+^ channels

**Figure 7:**
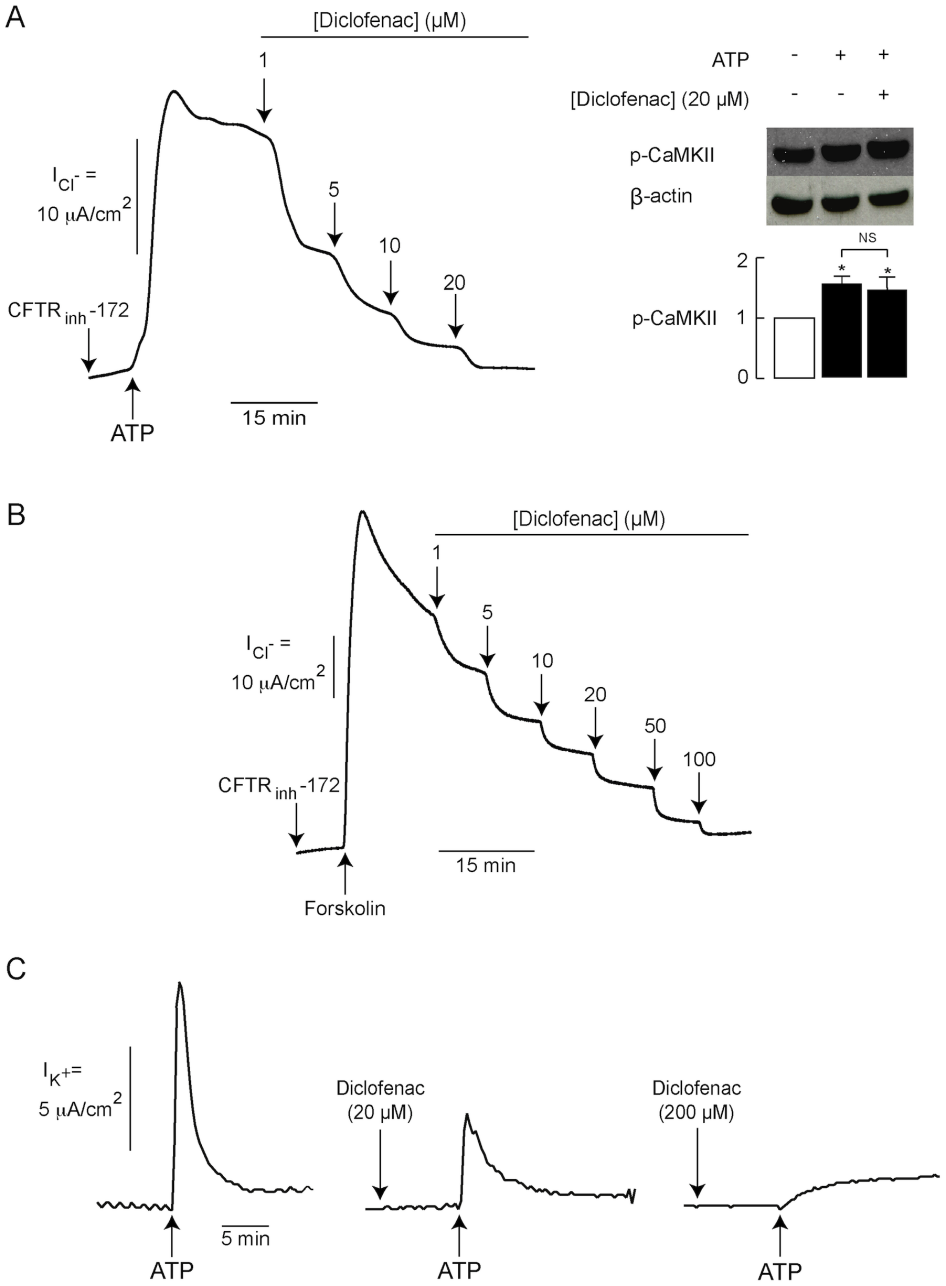
Effects of diclofenac on CaCC, IRC and Ca^2+^-activated basolateral K^+^ channel. (A) Inhibition of CaCC-mediated Cl^−^ transport by diclofenac. (left) Representative tracing of CaCC-mediated apical Cl^−^ current with basolateral membrane permeabilization. CFTR_inh_-172 (5 µM) and ATP (100 µM) were added into apical solutions before addition of diclofenac into both apical and basolateral solutions (n = 5). (right) Diclofenac had no effect on ATP-induced CaMKII phosphorylation. T84 cells were incubated for 20 min with vehicle (control), ATP (100 µM), or ATP (100 µM) plus diclofenac (20 µM). CaMKII phosphorylation was investigated using immunoblot analysis of phosphorylated CaMKII. Results of band intensity analysis are expressed as relative band intensity. NS, non-statistical difference; *, p<0.05 compared with control (n = 3). (B) Inhibition of IRC-mediated Cl^−^ transport by diclofenac. In this experiment, apical Cl^−^ current analysis was performed. CFTR_inh_-172 (5 µM) was added into apical solution before IRC activation by forskolin (20 µM). Diclofanac was added into both apical and basolateral solutions (n = 5). (C) Inhibition of Ca^2+^-activated basolateral K^+^ channel (K_Ca_3.1) by diclofenac. In this experiment, basolateral K^+^ current measurements were performed in the presence of BaCl_2_ (5 mM) in the apical solution. DMSO (control) or diclofenac was added into both apical and basolateral solutions before activation of K_Ca_3.1 by ATP (100 µM) (n = 4–6).

In addition to CFTR, Cl^−^ transport across apical membrane of T84 cells is mediated by two other types of Cl^−^ channels including Ca^2+^-activated Cl^−^ channel (CaCC) and inwardly rectifying Cl^−^ channel (IRC) [12]. We next determined the effects of diclofenac on these two apical Cl^−^ channels using apical Cl^−^ current analysis. To determine the effect of diclofenac on CaCC, T84 cell monolayers were pretreated with CFTR_inh_-172 (to prevent CFTR-mediated Cl^−^ transport) and CaCC-mediated apical Cl^−^ transport was stimulated by ATP (100 µM). As shown in Fig. 7A (left), diclofenac inhibited CaCC-mediated apical Cl^−^ current in a concentration-dependent manner with an IC_50_ of ∼1 µM and almost complete inhibition at 20 µM. Since the activation of CaCC is mediated by Ca^2+^/calmodulin-dependent protein kinase II (CaMKII) in T84 cells [30], we determined whether diclofenac interfered with the steps of ATP-induced CaMKII activation (starting from ATP-P2Y receptor binding to Ca^2+^ elevation-induced CaMKII phosphorylation) by performing immunoblot analysis of phosphorylated CaMKII, which is an indicator of CaMKII activation. As depicted in Fig. 7A (right), 20-min treatment with ATP (100 µM) induced CaMKII phosphorylation, which was unaffected by co-incubation with diclofenac (20 µM). The results suggest that the inhibition of CaCC-mediated apical Cl^−^ current by diclofenac is not due to the interference with ATP-induced CaMKII activation. Next, we determined the effect of diclofenac on IRC, a recently identified apical Cl^−^ channel activated via cAMP-exchange protein directly activated by cyclic AMP 1 (Epac1)-Ca^2+^-mediated pathways [7]. To measure IRC activity, T84 cells were pretreated with CFTR_inh_-172 (to exclude the contribution by CFTR) prior to IRC stimulation by forskolin. As depicted in Fig. 7B, IRC-mediated apical Cl^−^ current was concentration-dependently inhibited by diclofenac with an IC_50_ of ∼5 µM and near complete inhibition at 100 µM. Furthermore, the effect of diclofenac on Ca^2+^-activated basolateral K^+^ channels (i.e. K_Ca_3.1) was investigated using basolateral K^+^ current analysis in T84 cells. In this experiment, cells were pretreated with BaCl_2_ (5 mM; to prevent current contributed by cAMP-activated basolateral K^+^ channels) before stimulation of Ca^2+^-activated basolateral K^+^ channels by ATP (100 µM). As depicted in Fig. 7C, basolateral K^+^ current induced by ATP was dose-dependently inhibited by diclofenac with near complete inhibition at 200 µM. The results indicate that diclofenac suppressed the activities of CaCC, IRC and Ca^2+^-activated basolateral K^+^ channel in T84 cells.

### Evaluation of cytotoxic potential of diclofenac in T84 cells

**Figure 8:**
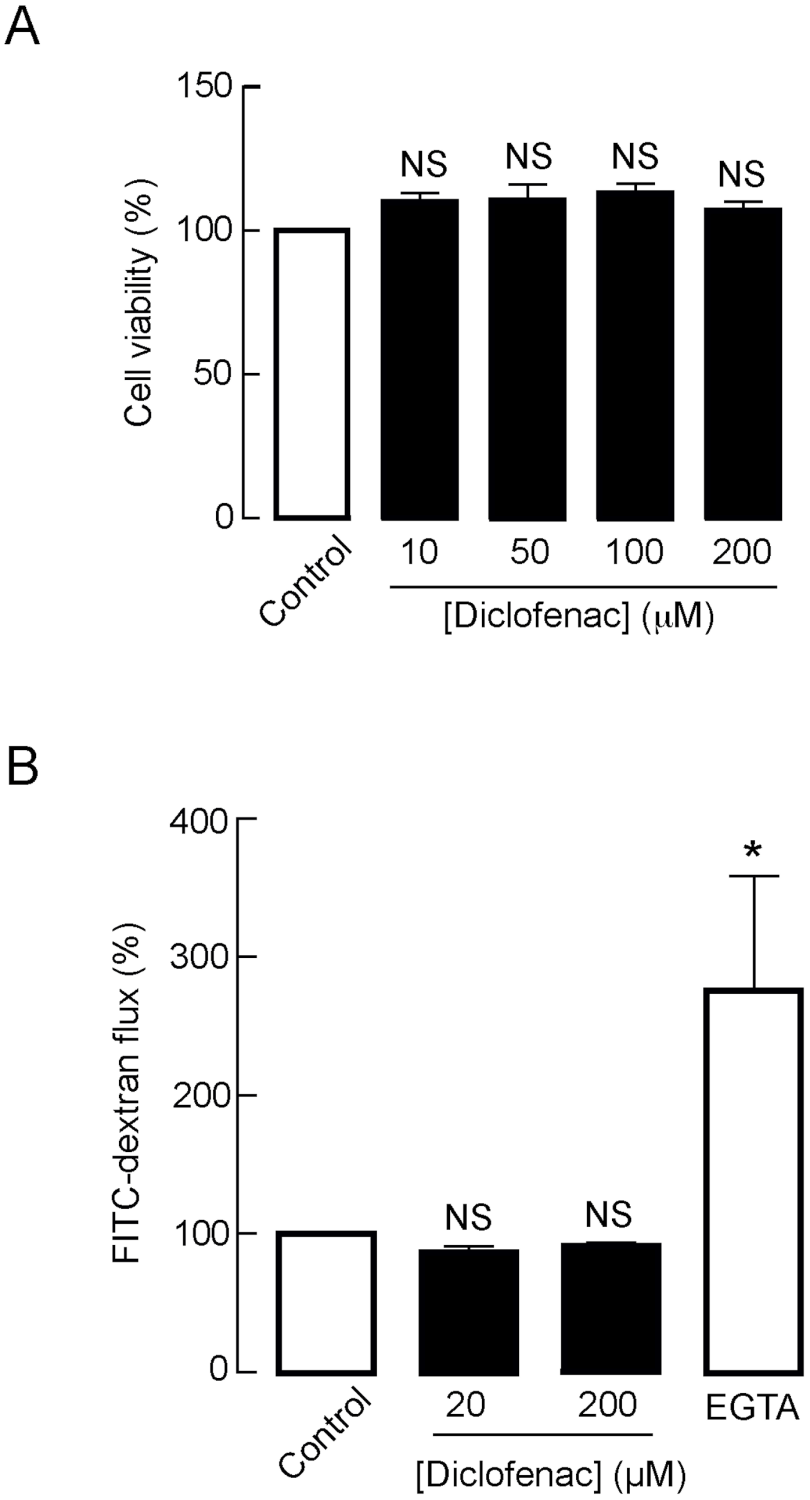
Evaluation of cytotoxic potential of diclofenac in T84 cells. (A) Effect of diclofenac on T84 cell viability. T84 cells grown in 96-well plates were incubated for 24 h with diclofenac at the indicated concentrations. MTT assays were used to determine T84 cell viability. Data are expressed as means of % control ± S.E.M. (n = 5). (B) Effect of diclofenac on barrier function. T84 cells grown on permeable support were exposed for 24 h to diclofenac at the indicated concentrations before measuring flux of FITC-dextran (molecular weight ∼4.4 kDa). Data are expressed as means of % control ± S.E.M. EGTA (3 mM) was used as a positive control. NS, non-statistical difference; *, p<0.05 compared with control (n = 4).

To evaluate potential intestinal toxicity of diclofenac, the effects of diclofenac on T84 cell viability and barrier function were investigated. Cell viability was determined by MTT assays. As illustrated in Fig. 8A, 24-h exposure to diclofenac, at concentrations from 10 µM to 200 µM, did not affect T84 cell viability. To assess the integrity of intestinal barrier function, flux of fluorescein isothiocyanate (FITC)-dextran (molecular weight 4.4 kDa), a fluorescence-tagged paracellular marker, across T84 cell monolayers was determined after 24-h treatment with diclofenac. As shown in Fig. 8B, diclofenac at concentrations of 20 µM and 200 µM did not change FITC-dextran flux across T84 cell monolayers compared with control. On the other hand, treatment with EGTA (3 mM), a Ca^2+^-chelating agent known to disrupt epithelial tight junctions, markedly increased the FITC-dextran flux serving as a positive control for this experiment. These experiments suggest that diclofenac, at concentrations found to inhibit transepithelial Cl^−^ secretion, is not cytotoxic to intestinal epithelial cells.

### Antidiarrheal application of diclofenac

**Figure 9:**
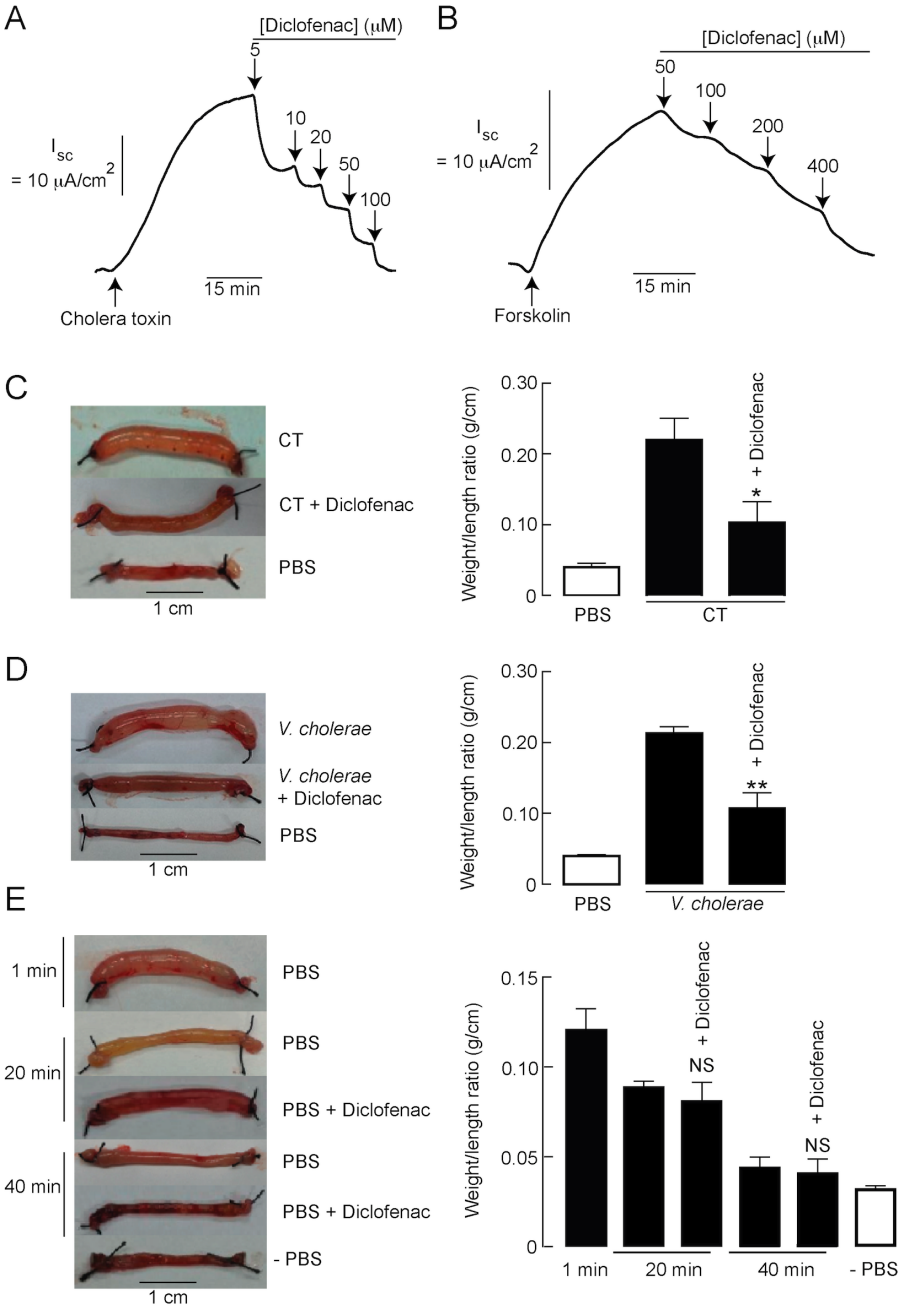
Antidiarrheal application of diclofenac. (A) Effect of diclofenac on cholera toxin (CT)-induced Cl^−^ secretion in T84 cells. Short-circuit current measurements were performed in T84 cells. After stimulation of Cl^−^ secretion by CT (1 µg/ml), diclofenac was added into both apical and basolateral solutions (n = 5). (B) Effect of diclofenac on cAMP-induced Cl^−^ secretion in mouse intestinal sheets. Mouse intestinal sheets were mounted in Ussing chambers and Cl^−^ secretion was stimulated by forskolin (20 µM). Diclofenac was added into both apical and basolateral solutions (n = 4). (C) Effect of diclofenac on CT-induced intestinal fluid secretion in mice. Ileal loops were instilled with PBS or PBS containing CT (1 µg/loop) with or without concomitant intraperitoneal administration of diclofenac (30 mg/kg). Four h later, ileal loops were removed for loop weight/length ratio measurements; (left) representative photographs of ileal loops, (right) summary of data. Data are expressed as means of loop weight/length ratio ± S.E.M. *, p<0.05 compared with CT-treated control (n = 8). (D) Effect of diclofenac on *V. cholerae*-induced intestinal fluid secretion in mice. Ileal loops were inoculated with PBS or PBS containing *V. cholerae* (10^7^ CFU/loop) with or without concomitant intraperitoneal administration of diclofenac (30 mg/kg). Twelve hours post-inoculation, ileal loops were removed for measurements of loop weight/length ratio; (left) representative photographs of ileal loops, (right) summary of data. Data are expressed as means of loop weight/length ratio ± S.E.M. **, p<0.01 compared with *V. cholerae*-inoculated control (n = 6) (E) Effect of diclofenac on intestinal fluid absorption. Ileal loops were instilled with PBS with or without intraperitoneal administration of diclofenac (30 mg/kg). Twenty or forty min later, ileal loops were removed for loop weight/length ratio measurements; (left) representative photographs of ileal loops, (right) summary of data. Ileal loops at 1 min after PBS instillation and ileal loops without PBS instillation (-PBS) were shown for comparisons. Data are expressed as means of loop weight/length ratio ± S.E.M. (n = 6–8). NS, non-statistical difference compared with control at the same time point.

Since cAMP-induced Cl^−^ secretion plays an important role in the pathogenesis of secretory diarrheas especially cholera [12], we investigated the potential application of diclofenac in the treatment of cholera using both *in vitro* and *in vivo* models. As demonstrated in Fig. 9A, diclofenac inhibited cholera toxin (CT)-induced Cl^−^ secretion in T84 cells with an IC_50_ of ∼10 µM and >95% inhibition at 100 µM. Likewise, diclofenac inhibited forskolin-induced Cl^−^ secretion in mouse intestinal sheets, although with lower potency compared to the studies in T84 cells (Fig. 9B). Of particular importance, antidiarrheal efficacy of diclofenac was investigated in mouse closed-loop models of cholera induced by either CT or *V. cholerae*. In this experiment, loop weight/length ratio was used as an indicator of intestinal fluid secretion and diclofenac was intraperitoneally administered at a dose of 30 mg/kg. Based on the principle of body surface area-based dosage conversion [31], this dose of diclofenac in mice is equivalent to 2 mg/kg of diclofenac in human, which is the dose for treatments of pain and inflammation. Interestingly, concomitant intraperitoneal administration of diclofenac (30 mg/kg) significantly inhibited both CT- and *V. cholerae*-induced fluid secretion by ∼70% (Fig. 9C and Fig. 9D). Further, the effect of diclofenac alone on intestinal fluid absorption was determined using a mouse closed-loop model. In this experiment, closed ileal loops were instilled with PBS with or without intraperitoneal administration of diclofenac (30 mg/kg) and, 20 min or 40 min later, the ileal loops were removed for loop weight/length ratio measurements. As shown in Fig. 9E, the ileal loop weight/length ratio of diclofenac-treated groups was not significantly different from that of control at both 20 min and 40 min after PBS instillation, indicating that diclofenac had no effect on intestinal fluid absorption. Data of ileal loops at 1 min after PBS instillation (without diclofenac treatment) and ileal loops without PBS instillation (-PBS, empty loop) were included for comparisons.

## Discussion

In this report, we demonstrated the inhibitory effect of diclofenac, a commonly used NSAID, on cAMP-activated Cl^−^ secretion in human intestinal epithelial cell line (T84 cells). Functional analyses of membrane transport processes involved in Cl^−^ secretion indicate that the effect of diclofenac involves inhibition of CFTR Cl^−^ channels and cAMP-activated basolateral K^+^ channels. Interestingly, diclofenac also inhibited two other types of apical Cl^−^ channels, namely, Ca^2+^-activated Cl^−^ channels (CaCC) and inwardly rectifying Cl^−^ channels (IRC), and suppressed Ca^2+^-activated basolateral K^+^ channels T84 cells. More importantly, this study revealed a potential utility of diclofenac in the treatment of cholera using both *in vitro* and *in vivo* models.

Cyclic AMP-activated Cl^−^ secretion by enterocytes requires coordinated functions of several transport proteins located in both apical and basolateral membranes. Chloride anion is uptaken into enterocytes via Na^+^-K^+^-Cl^−^ cotransporters and subsequently transported into intestinal lumen via cAMP-activated apical Cl^−^ channels, namely CFTR and IRC. However, our previous and present studies (Fig. 1B) indicate that cAMP-activated apical Cl^−^ efflux is mainly via CFTR, and that IRC plays a significant role only after inhibition of CFTR [15]. Additional transport proteins required for cAMP-activated Cl^−^ secretion are cAMP-activated basolateral K^+^ channels and Na^+^-K^+^-ATPases, which are required for recycling K^+^ and Na^+^ back to serosa, respectively, and thus play crucial roles in maintaining electrochemical driving force for apical Cl^−^ efflux into intestinal lumen. Inhibition of one of these transport proteins can stop the whole process of cAMP-activated Cl^−^ secretion [4], [32], [33]. Because inhibition of cAMP-activated Cl^−^ secretion was observed when diclofenac was added into either apical or basolateral solutions, we hypothesized that diclofenac may target either apical or basolateral transport proteins. In order to prove this hypothesis, the effects of diclofenac on functions of individual transport proteins (i.e. CFTR, cAMP-activated basolateral K^+^ channel, Na^+^-K^+^ ATPase and NKCC1) were investigated.

The underlying mechanisms of diclofenac inhibition of CFTR-mediated Cl^−^ transport were investigated in T84 cells using apical Cl^−^ current measurements. Results showed that IC_50_ of diclofenac was ∼8 µM–10 µM regardless of the mechanisms of CFTR activation (increases in cAMP levels by forskolin, direct stimulation of PKA by CPT-cAMP, or direct CFTR activation by genistein). In agreement with this result, we found that the inhibitory effect of diclofenac on CFTR-mediated apical Cl^−^ current was unaffected by pharmacological inhibition of PDE, MRP4, protein phosphatase or AMPK, all of which are negative regulators of CFTR Cl^−^ channel activity. In addition, the levels of intracellular cAMP were unaffected by diclofenac. These results indicate that, in T84 cells, the inhibition of CFTR by diclofenac is not via indirect mechanisms including decreasing cAMP levels (by inhibition of adenylate cyclase or activation of PDE or MRP4), dephosphorylation of CFTR (by protein phosphatase), or phosphorylation by AMPK. We speculate that diclofenac may inhibit CFTR Cl^−^ channel activity by acting directly on the channel. Interestingly, the inhibitory effect of diclofenac on CFTR function was also observed in Calu-3 cells, human airway epithelial cells endogenously expressing CFTR, suggesting that the effect of diclofenac is not cell line-specific.

In addition to the inhibitory effect on CFTR, diclofenac blocked the cAMP-activated basolateral K^+^ channels in T84 cells. Of note, the potency of diclofenac on the inhibition of cAMP-activated Cl^−^ secretion (IC_50_∼20 µM) is lower than that on the inhibition of CFTR (IC_50_∼10 µM) and cAMP-activated basolateral K^+^ channels (IC_50_∼3 µM). This may be due to the intracellular negative membrane potential which impedes the entry of negatively charged diclofenac into the cells, resulting in lower intracellular concentration of diclofenac in intact cells (in short-circuit current analysis) than in permeabilized cells (in apical Cl^−^ and basolateral K^+^ current analysis; membrane potential is ∼0 mV) at any given concentrations of diclofenac in bathing solutions. Furthermore, we found that diclofenac inhibited other types of ion channels involved in intestinal Cl^−^ secretion including IRC, CaCC and Ca^2+^-activated basolateral K^+^ channels without affecting Na^+^-K^+^ ATPase and NKCC1 activities. Therefore, all of the data obtained from functional analysis of individual transport proteins indicate that diclofenac inhibits Cl^−^ secretion mediated by both cAMP and Ca^2+^-dependent pathways. Of particular interest, diclofenac inhibited ATP-induced CaCC-mediated Cl^−^ transport without any effects on ATP-induced CaMKII phosphorylation, indicating that diclofenac may directly inhibit CaCC. Indeed, CaCC-mediated Cl^−^ secretion by enterocytes plays pivotal roles in driving intestinal fluid secretion in rotavirus diarrhea, the most common cause of infectious diarrhea in children under 5 years of age [34], [35]. Accordingly, diclofenac or related compounds may be of particular benefit in the treatment of rotavirus diarrhea. Furthermore, since clinical use of diclofenac is known to be associated with constipation [36], [37], our findings may provide a mechanistic insight into the cellular events underlying constipation in patients taking diclofenac.

To date, several classes of potential antidiarrheal therapeutics for cholera have been identified, with CFTR inhibitor being recognized as the most promising candidate [12]. In support of this notion, CFTR_inh_-172, a small-molecule CFTR inhibitor identified by high-throughput screening, has been shown to reduce both cholera toxin (CT)- and live *V. cholerae*-induced intestinal fluid secretion in mice by >90% [17], [38]. Until now, several classes of CFTR inhibitors have been identified and shown to exhibit antidiarrheal efficacy in animal models of cholera [12], [14]–[16]. However, the development of these CFTR inhibitors into new antidiarrheal therapy has progressed slowly, probably, due to the limited financial incentives for the investment in research and development of drugs for cholera, which is prevalent in developing countries. Therefore, it may be more reasonable to develop antidiarrheal therapy of cholera by extending clinical applications of known drugs that are found to inhibit CFTR-mediated Cl^−^ secretion. In the present study, we found that diclofenac effectively abrogated CT-induced Cl^−^ secretion in T84 cells with an IC_50_ of ∼10 µM, which is lower than its potency on the inhibition of Cl^−^ secretion induced by other CFTR agonists including forskolin, CPT-cAMP and genistein. Higher potency on the inhibition of Cl^−^ secretion induced by CT compared to other CFTR agonists indicates that diclofenac may have other beneficial pleiotropic effects against CT intoxication in T84 cells. Importantly, we demonstrated that diclofenac at a dose of 30 mg/kg inhibited CT- and *V. cholerae*-induced intestinal fluid secretion by 70% in mouse closed-loop models. Based on the body surface areas of mice and humans [31], this dose of diclofenac (30 mg/kg) could be converted into the human equivalent dose of ∼2 mg/kg, which is the dose recommended for treatments of pain and inflammation in human. Furthermore, diclofenac had no cytotoxicity in T84 cells, as revealed by cell viability assay and measurements of barrier function, and had no effects on basal intestinal fluid absorption, both of which are prerequisite properties of an antidiarrheal therapy. These results indicate that diclofenac represent a class of known drug that may have potential utility in the treatment of cholera. Future studies will be required to determine antidiarrheal efficacy of diclofenac in the treatment of cholera in humans.

**Figure 10:**
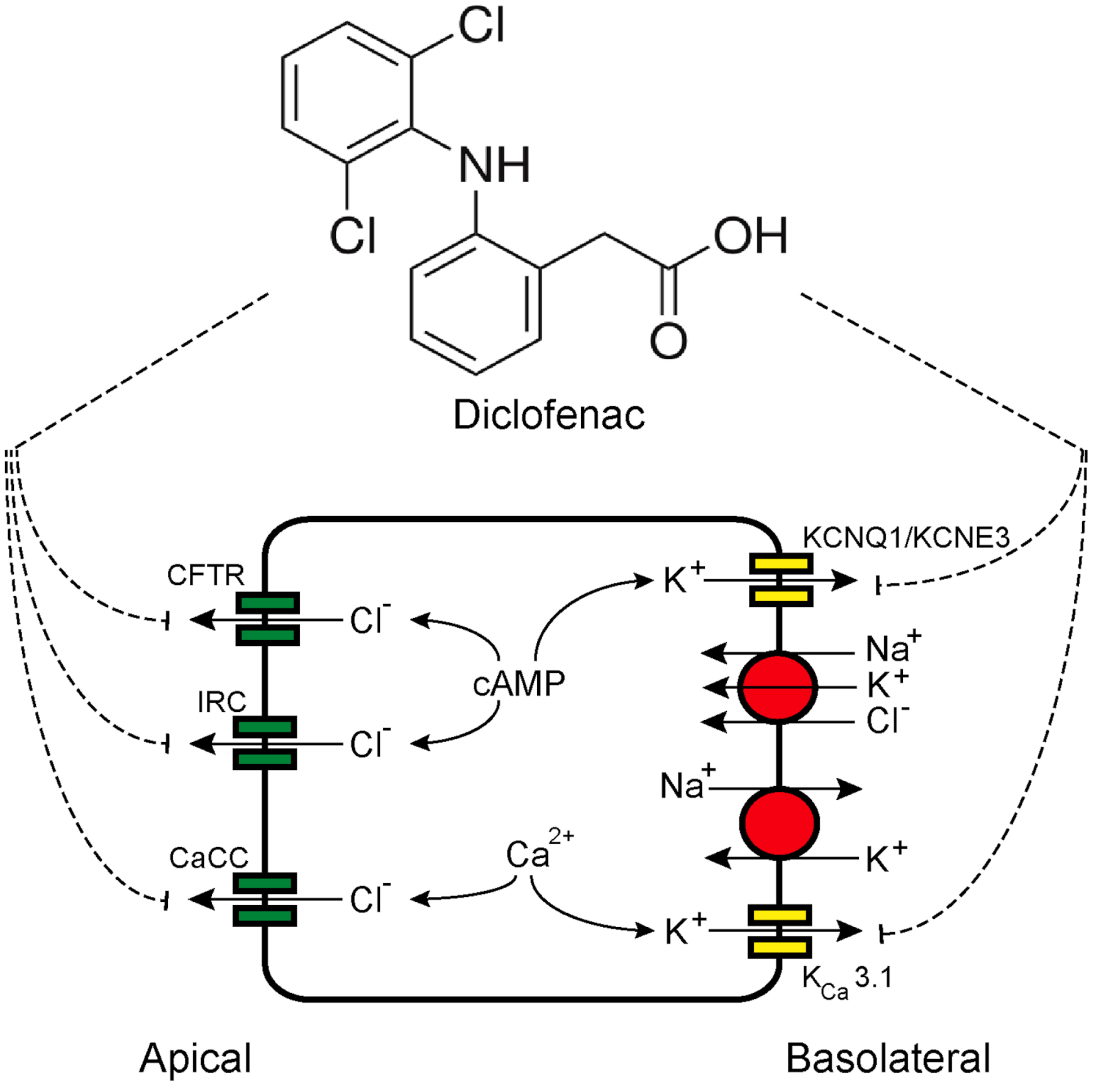
Proposed mechanisms of inhibition of transepithelial Cl^−^ secretion by diclofenac in T84 cells.

In conclusion, this study reveals diclofenac as an inhibitor of Cl^−^ secretion across human intestinal epithelial cells. The mechanisms of inhibition involve blockades of apical Cl^−^ channels (CFTR, CaCC and IRC) and basolateral K^+^ channels (KCNQ1/KCNE3 and K_Ca_3.1) (Fig. 10). Our findings may lead to the successful development of diclofenac or related compounds into an inexpensive and effective therapy of secretory diarrheas resulting from either cAMP or Ca^2+^-activated Cl^−^ secretion including cholera and rotavirus diarrheas.
